# Supplementary material for: Splice-Junction-Based Mapping of Alternative Isoforms in the Human Proteome
Source: Cell Rep. Author manuscript; Available in PMC 2020 Jan 15. (PMC6961840; doi:10.1016/j.celrep.2019.11.026)
Supplement: 2 [file NIHMS1546469-supplement-2.zip › DF1/PXD009737/TestisTrypsin/TestisTrypsin_32_COPE_QPSGGSGEVDELFDVK.pdf]

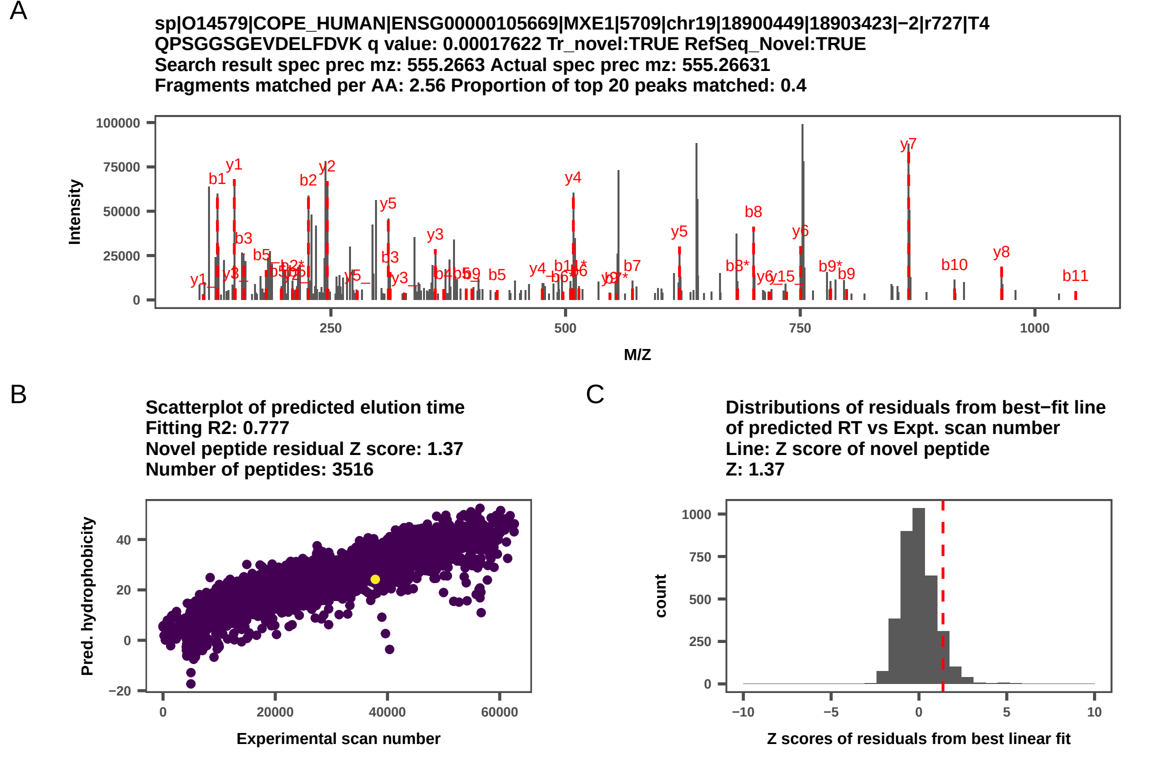
A
sp|O14579|COPE_HUMAN|ENSG00000105669|MXE1|5709|chr19|18900449|18903423|−2|r727|T4
QPSGGSGEVDELFDVK q value: 0.00017622 Tr_novel:TRUE RefSeq_Novel:TRUE
Search result spec prec mz: 555.2663 Actual spec prec mz: 555.26631
Fragments matched per AA: 2.56 Proportion of top 20 peaks matched: 0.4
Intensity
100000
75000
50000
25000
0
250
500
750
1000
M/Z
B
Scatterplot of predicted elution time
Fitting R2: 0.777
Novel peptide residual Z score: 1.37
Number of peptides: 3516
Pred. hydrophobicity
40
20
0
−20
0
20000
40000
60000
Experimental scan number
C
Distributions of residuals from best−fit line
of predicted RT vs Expt. scan number
Line: Z score of novel peptide
Z: 1.37
count
1000
750
500
250
0
−10
−5
0
5
10
Z scores of residuals from best linear fit
